# Full-length ATP7B reconstituted through protein *trans*-splicing corrects Wilson disease in mice

**DOI:** 10.1016/j.omtm.2022.08.004

**Published:** 2022-08-13

**Authors:** Agnese Padula, Raffaella Petruzzelli, Sasha A. Philbert, Stephanie J. Church, Federica Esposito, Severo Campione, Marcello Monti, Filomena Capolongo, Claudia Perna, Edoardo Nusco, Hartmut H. Schmidt, Alberto Auricchio, Garth J.S. Cooper, Roman Polishchuk, Pasquale Piccolo

**Affiliations:** 1 Telethon Institute of Genetics and Medicine, Pozzuoli, Italy; 2 Scuola Superiore Meridionale, University of Naples Federico II, Naples, Italy; 3 Division of Cardiovascular Sciences, School of Medical Sciences, Faculty of Biology, Medicine and Health, University of Manchester, Manchester, UK; 4 Centre for Advanced Discovery and Experimental Therapeutics (CADET), Manchester Academic Health Sciences Centre, Manchester, UK; 5 Pathology Unit, A. Cardarelli Hospital, Naples, Italy; 6 Department of Gastroenterology and Hepatology, University Hospital Duisburg-Essen, Essen, Germany; 7 Department of Advanced Biomedical Sciences, University of Naples Federico II, Naples, Italy; 8 School of Biological Sciences, Faculty of Science, University of Auckland, Auckland, New Zealand

**Keywords:** adeno-associated vectors, split inteins, protein *trans*-splicing, Wilson disease, copper storage

## Abstract

Wilson disease (WD) is a genetic disorder of copper homeostasis, caused by deficiency of the copper transporter ATP7B. Gene therapy with recombinant adeno-associated vectors (AAV) holds promises for WD treatment. However, the full-length human *ATP7B* gene exceeds the limited AAV cargo capacity, hampering the applicability of AAV in this disease context. To overcome this limitation, we designed a dual AAV vector approach using split intein technology. Split inteins catalyze seamless ligation of two separate polypeptides in a highly specific manner. We selected a DnaE intein from *Nostoc punctiforme* (Npu) that recognizes a specific tripeptide in the human *ATP7B* coding sequence. We generated two AAVs expressing either the 5′-half of a codon-optimized human *ATP7B* cDNA followed by the N-terminal Npu DnaE intein or the C-terminal Npu DnaE intein followed by the 3′-half of *ATP7B* cDNA, under the control of a liver-specific promoter. Intravenous co-injection of the two vectors in wild-type and *Atp7b*^−/−^ mice resulted in efficient reconstitution of full-length ATP7B protein in the liver. Moreover, *Atp7b*^−/−^ mice treated with intein-ATP7B vectors were protected from liver damage and showed improvements in copper homeostasis. Taken together, these data demonstrate the efficacy of split intein technology to drive the reconstitution of full-length human ATP7B and to rescue copper-mediated liver damage in *Atp7b*^−/−^ mice, paving the way to the development of a new gene therapy approach for WD.

## Graphical abstract

**Figure:**
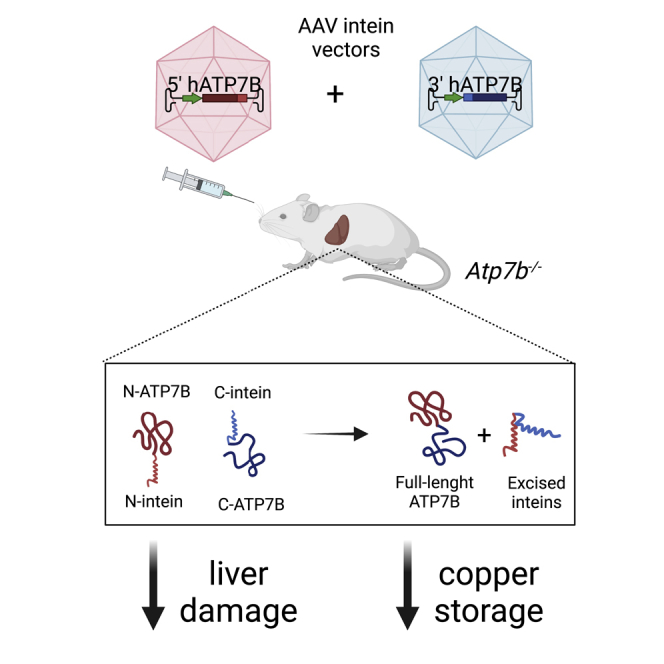

## Teaser

Wilson disease is an inherited disorder of copper metabolism due to mutations in ATP7B and is an attractive target for gene therapy. However, ATP7B size surpasses adeno-associated viral vector capacity. Piccolo and colleagues applied a dual-vector strategy exploiting split intein to reconstitute full-length ATP7B by protein *trans*-splicing, achieving significant disease amelioration.

## Introduction

Wilson disease (WD, OMIM: 277900) is a life-threating rare autosomal disorder of copper homeostasis, caused by pathogenic variants in the *ATP7B* gene, encoding for a P-type copper transporting ATPase mainly expressed in hepatocytes.^1^^,^^2^ ATP7B exerts a key role in copper metabolism, providing copper for cuproprotein synthesis and releasing excessive copper into the bile.^3^^,^^4^ Lack of functional ATP7B results in toxic copper accumulation and severe progressive liver and brain diseases.^5^^,^^6^ Current therapies for WD are based on copper chelators and zinc salts to reduce tissue copper storages and intestinal absorption. Although such treatments provide clinical benefits to most patients,^5^ they are not effective in all WD patients, and non-responders usually require liver transplantation.^7^ Moreover, compliance to treatments is often problematic, especially in adolescents, due to pharmacological side effects and treatment duration.^8^^,^^9^ Thus, alternative and more definitive therapies are highly desirable.

Liver-directed gene therapy holds great potential for the treatment of WD, and adeno-associated vectors (AAV) are the most promising gene transfer platforms based on their safety and efficacy in humans. A previous study has shown that AAV-mediated delivery of full-length human ATP7B to *Atp7b*^*−/−*^ livers achieved correction of copper metabolism.^10^ However, the large size of *ATP7B* cDNA surpasses the AAV cargo capacity, resulting in an oversize vector genome, poor vector production yields, and lack of efficacy in female and old male *Atp7b*^*−/−*^ mice.^10^ More recently, delivery of a mini-ATP7B, with four out of six metal binding domains deleted, was found to be more effective because it restored copper homeostasis in male and female *Atp7b*^−/−^ mice.^11^ However, although MBD1–4 are not essential for ATP7B function,^12^ copper transport activity by mini-ATP7B appeared to be reduced compared with full-length ATP7B.^13^

To overcome the ATP7B size limitation, we investigated an alternative approach based on dual AAV vectors and split intein technology. Split inteins are a subset of inteins that catalyze seamless ligation of two separate polypeptides in a highly specific manner, resulting in generation of a single, larger polypeptide.^14^ By this approach, a protein encoding sequence is split in two or more halves, each associated to sequences encoding split inteins (N-intein and C-intein). Once each split intein encounters its complementary partner, the reconstituted intein excises itself from the host protein while mediating ligation of the N- and C-polypeptides via a peptide bond.^15^^,^^16^ In the present work, we evaluated whether full-length ATP7B in hepatocytes can be reconstituted by split intein technology and its efficacy in restoring copper homeostasis and prevention of liver disease of a WD mouse model.

## Results

### Full-length ATP7B reconstitution by intein-mediated protein *trans*-splicing

**Figure 1:**
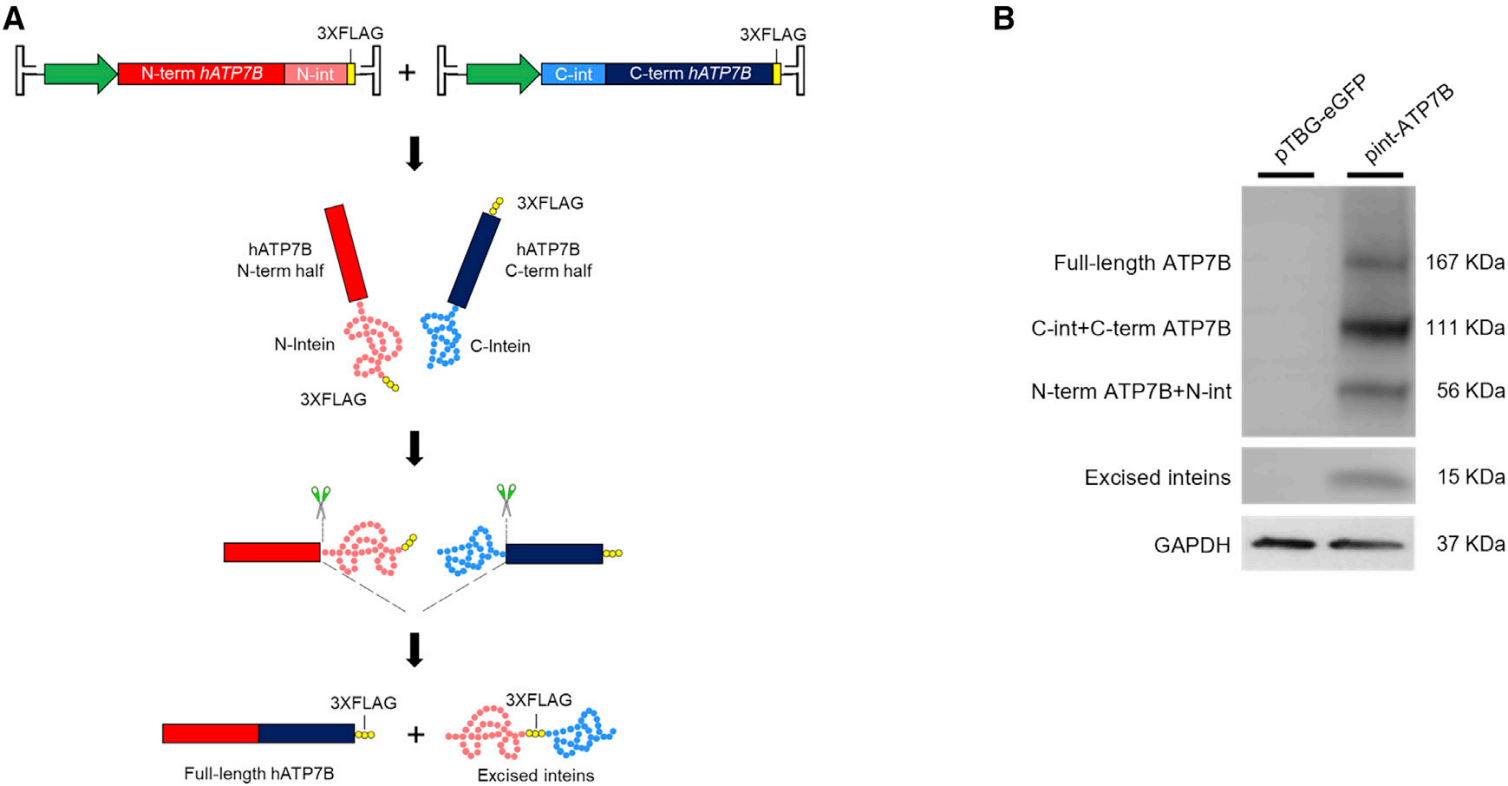
Split-intein-driven reconstitution of full-length human ATP7B (A) Schematic representation of AAV intein-mediated protein *trans*-splicing. (B) Western blot analysis on extracts from ATP7B-KO HepG2 cells co-transfected with int-ATP7B constructs (pint-ATP7B) or with a control GFP plasmid (pTBG-EGFP) using anti-FLAG antibody. Expected molecular weights are 167 kDa for full-length ATP7B-3XFLAG, 111 kDa for C-intein-C-term ATP7B half-3XFLAG, 56 kDa for N-term ATP7B half-N-intein-3XFLAG, and 15 kDa for excised inteins. GAPDH was used as a loading control.

DNA polymerase III (DnaE) from *Nostoc punctiforme* (*Npu*) catalyzes rapid protein *trans*-splicing and has shown tolerance for extein sequence variation^17^ and minimal sensitivity to the nature of N-extein residues.^18^ C-extein sequence requirements are limited to catalytic Cys at the +1 position and large hydrophobic residues are preferred at the +2 position,^18^ with Phe residue in the +2 position resulting in highest splicing efficiency.^19^ The human ATP7B protein sequence presents a Cys-Phe dipeptide at position 490–491, preceded by Pro, Gln, and Lys residues, which are permissive for DnaE intein-driven protein *trans*-splicing.^18^ Selecting Cys^490^ as the splitting point resulted in cDNA sequences encoding for the N- and C-terminal ATP7B halves with respective lengths of 1,467 bp and 2,931 bp, which were compatible with AAV packaging requirements. We generated two constructs, each bearing either the N- or the C-terminal half of a codon-optimized human *ATP7B* cDNA fused to the N- and C-terminal halves of the *Npu* DnaE split intein (Figure 1A), respectively.^20^^,^^21^ Each intein-ATP7B construct included a triple FLAG tag (3xFLAG) for detection of both halves, full-length reconstituted ATP7B, and spliced inteins (Figure 1A).^22^ Intein-ATP7B constructs (int-ATP7B) were co-transfected in HepG2 knocked-out for *ATP7B* (ATP7B-KO) to evaluate their ability to reconstitute full-length ATP7B.^23^ Bands at the expected sizes for full-length ATP7B protein (167 kDa) and excised inteins (15 kDa) were detected by western blot analysis using anti-FLAG antibody (Figure 1B). Unspliced ATP7B halves fused to the N- and C-inteins (56 and 111 kDa, respectively) were also detected (Figure 1B).

### Intein-reconstituted ATP7B is functional and displays normal intracellular trafficking and function

**Figure 2:**
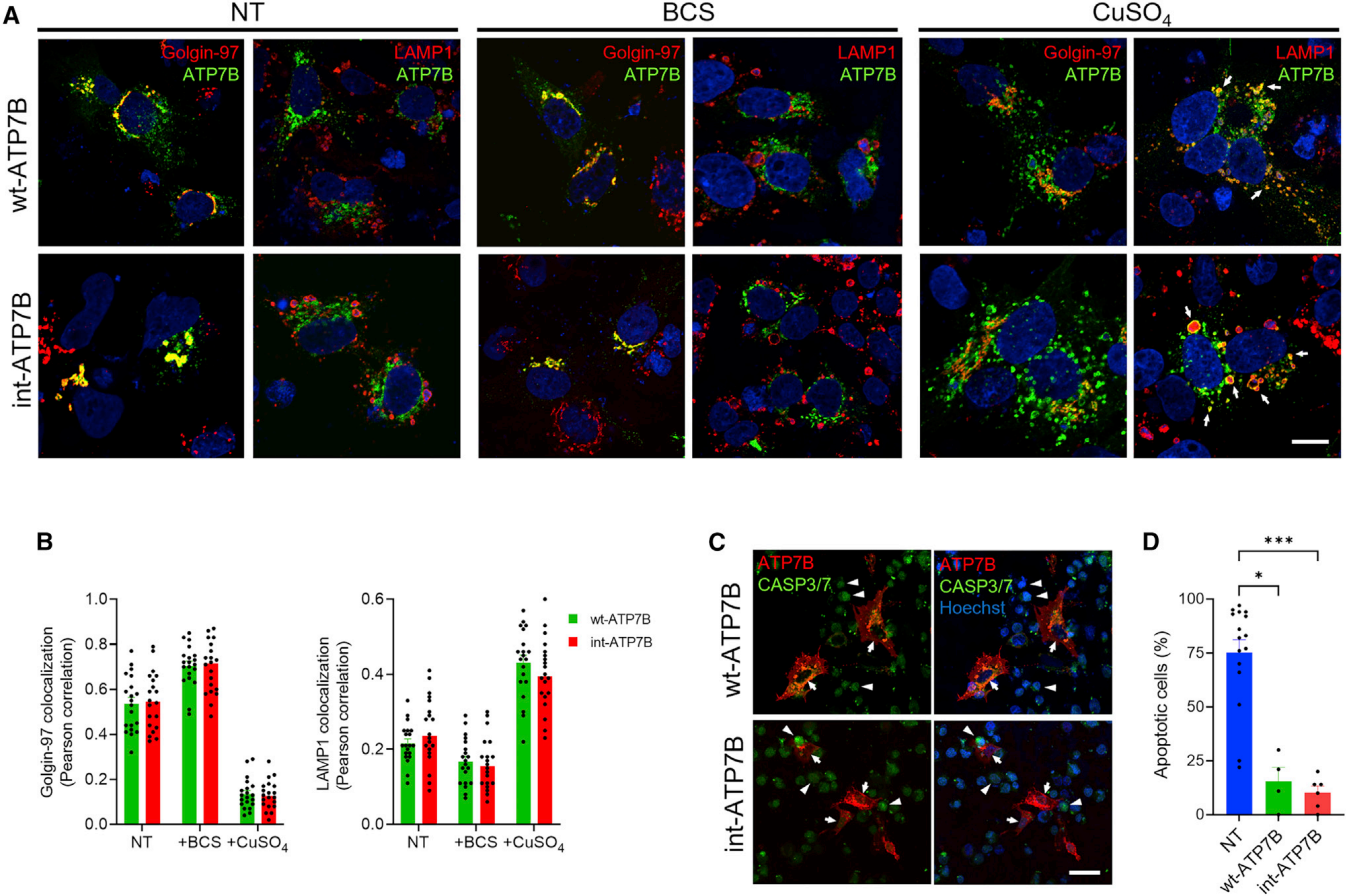
Intracellular trafficking and functioning of intein-reconstituted ATP7B (A) Representative images from immunofluorescent staining of ATP7B-KO HepG2 cells transfected with wt-ATP7B or int-ATP7B constructs and left untreated (left panels), or after treatment with copper chelator bathocuproine disulfonate (BCS, center panels) or with CuSO_4_ (right panels). Cells were labeled with antibodies against Golgin-97 and LAMP1 (red), and anti-ATP7B or anti-FLAG antibody to detect ATP7B (green). Arrows indicate LAMP1-positive structures containing ATP7B. Scale bar: 10 μm. (B) Golgin-97 and LAMP1 colocalization analysis (Pearson’s coefficient) with ATP7B. (C) Representative images of ATP7B-KO HepG2 cells transfected with wild-type ATP7B, or int-ATP7B constructs, or non-transfected and then treated with CuCl_2_. Cells were stained with CellEvent Caspase-3/7 detection reagent (CASP-3/7, green) and anti-ATP7B or anti-FLAG antibody to detect ATP7B (red). Arrows and triangles indicate ATP7B-positive/CASP-3/7-negative and ATP7B-negative/CASP-3/7-positive cells, respectively. Scale bar: 30 μm. (D) Quantification of CASP-3/7-positive cells per total nuclei. One-way ANOVA and Kruskal-Wallis post-hoc test: ∗p < 0.05; ∗∗∗p < 0.001. Data are shown as average ± SEM.

To investigate intracellular localization and trafficking of intein-reconstituted ATP7B, wild-type human ATP7B (wt-ATP7B) and int-ATP7B expressing plasmids were transfected in ATP7B-KO cells treated with copper-chelating agent bathocuproine disulfonate (BCS) to mimic copper deprivation, with CuSO_4_, or left untreated. Similar to wt-ATP7B, intein-reconstituted ATP7B overlapped with the *trans*-Golgi marker network Golgin-97 at normal and low copper concentrations, and it relocated to Lamp1-positive late endosomes/lysosomes after CuSO_4_ challenge (Figures 2A, 2B, and S1). Next, we investigated the function of intein-reconstituted ATP7B by testing copper resistance in ATP7B-KO cells. Cells were treated with CuCl_2_ and transfected with wt-ATP7B and int-ATP7B. Quantification of apoptotic nuclei showed up to 80% mortality in non-transfected ATP7B-KO cells, which was strongly reduced by both wt-ATP7B and int-ATP7B constructs (Figures 2C and 2D). Taken together, these findings show that split intein technology drives reconstitution of functional full-length ATP7B.

### AAV-mediated int-ATP7B gene therapy prevents liver damage and improves copper homeostasis in WD mice

**Figure 3:**
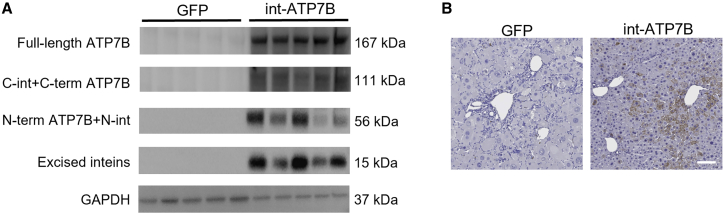
Robust intein-mediated reconstitution of full-length human ATP7B in *Atp7b*^*−/−*^ mice (A) Western blot analysis using anti-FLAG antibody of whole liver lysate from *Atp7b*^−/−^ mice injected with AAV2/8 TBG eGFP (GFP) at a dose of 2×10^13^ gc/Kg or co-injected with AAV2/8-HLP-5′ATP7B-N-intein and AAV2/8-HLP-C-intein-3′ATP7B (int-ATP7B), at a total dose of 2×10^13^ gc/Kg. Expected molecular weights are 167 kDa for full-length ATP7B-3XFLAG, 111 kDa for C-intein-c-term ATP7B half-3XFLAG, 56 kDa for N-term ATP7B half-N-intein-3XFLAG, and 15 kDa for excised inteins. GAPDH was used as loading control. (B) Representative images from immunohistochemistry using anti-ATP7B antibody on livers from *Atp7b*^−/−^ mice injected with GFP or int-ATP7B vectors. (n = 3 per group; Scale bar: 100 μm).

We generated AAV2/8 vectors carrying int-ATP7B constructs or a human full-length ATP7B cDNA, under the control of the hybrid liver-specific enhancer/promoter (HLP).^22^ Inclusion of the short HLP promoter resulted in vector genome sizes that were within the AAV packaging capacity for both int-ATP7B constructs (3.5 kb for AAV2/8-HLP-5′ATP7B-N-intein and 4.7 kb for AAV2/8-HLP-C-intein-3′ATP7B), while AAV2/8-HLP-full-lengthATP7B genome slightly exceeded AAV packaging capacity (5.0 kb). Southern blot analysis of AAV vector preparation including full-length ATP7B showed several bands with a molecular weight <5.0 kb, indicating packaging of truncated vector genomes (Figure S2). Conversely, aberrantly packaged genomes were not detected in int-ATP7B vector preparations (Figure S2). Int-ATP7B AAV vectors were intravenously co-injected at a total dose of 2×10^13^ gc/Kg in C57BL/6 wild-type mice to investigate ATP7B reconstitution *in vivo*. Western blot analysis using anti-FLAG antibody on liver lysates showed reconstituted full-length ATP7B, together with excised inteins and unspliced intein-ATP7B halves (Figure S3). To investigate the efficacy of intein-mediated gene therapy for WD, we used *Atp7b*^−/−^ mice that recapitulate several features of WD and are useful to investigate WD therapies.^10^^,^^11^ In *Atp7b*^−/−^ mice, copper starts to accumulate in the liver during the first weeks of life, leading to overt liver disorder by 12–20 weeks of age.^24^ We co-injected int-ATP7B vectors intra-venously in 6- to 7-week-old *Atp7b*^−/−^ mice at a total dose of 5×10^12^ or 2×10^13^ gc/Kg. An AAV-TBG-GFP vector (GFP) was injected as control for the injection at the dose of 2×10^13^ gc/Kg. Mice were sacrificed at 12 weeks post-injection, and western blot analysis using anti-FLAG antibody on liver lysates showed robust reconstituted full-length ATP7B, together with excised inteins and non-spliced intein-ATP7B halves in *Atp7b*^−/−^ mice injected with int-ATP7B vectors (Figure 3A). Consistently, immunohistochemistry using anti-ATP7B antibody showed ATP7B expression in hepatocytes, while no signal was detected in section from GFP-treated control *Atp7b*^−/−^ mice (Figure 3B).

**Figure 4:**
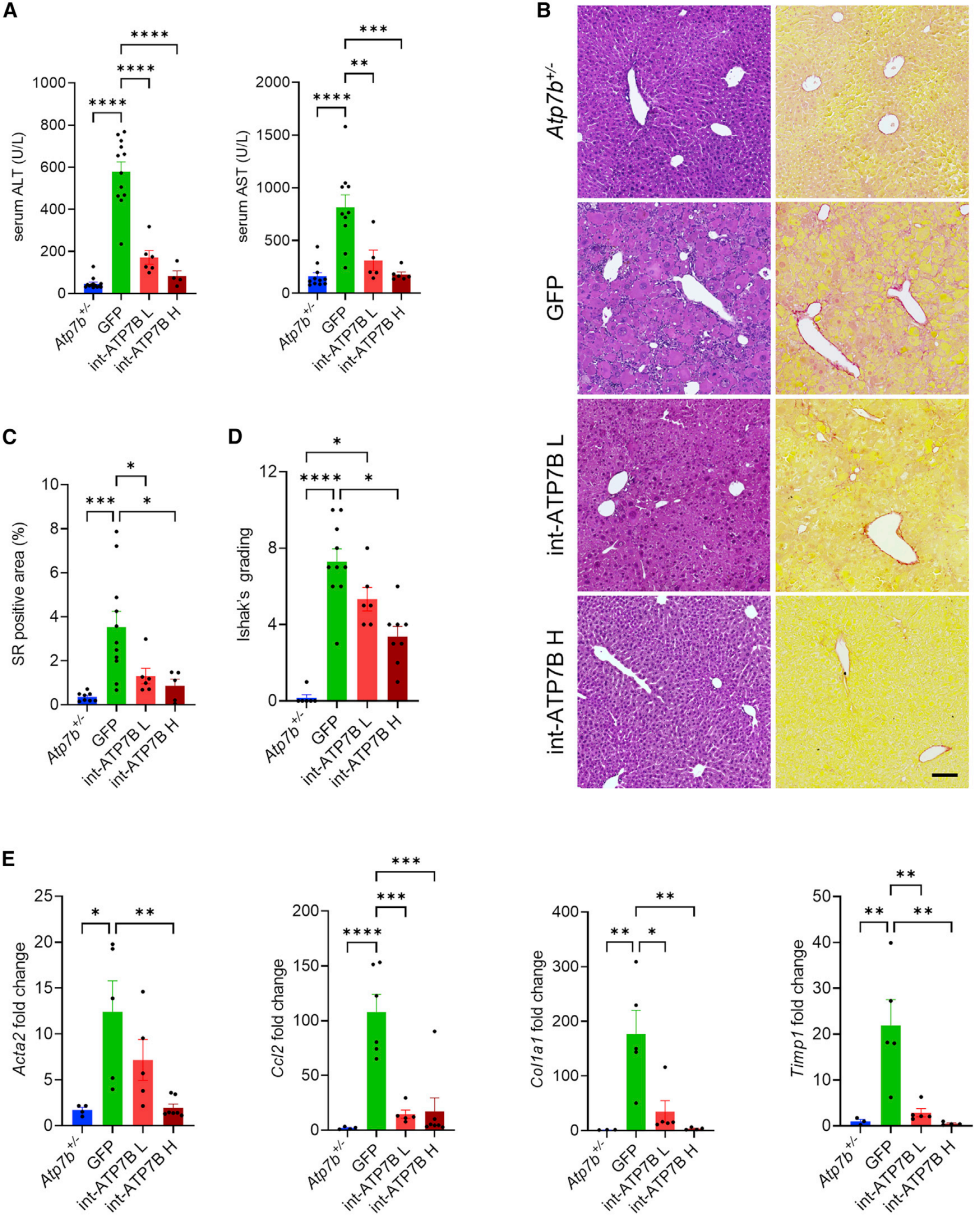
Intein-mediated gene therapy effect on preventing hepatocellular damage and liver disease progression in *Atp7b*^*−/−*^ mice (A) Serum alanine and aspartate aminotransferase (ALT and AST) levels in *Atp7b*^+/−^ healthy control mice and *Atp7b*^−/−^ mice injected with AAV2/8-TBG-eGFP (GFP) or AAV2/8-HLP-5′ATP7B-N-intein and AAV2/8-HLP-C-intein-3′ATP7B at total doses of 5×10^12^ (int-ATP7B L) or 2×10^13^ gc/kg (int-ATP7B H). (B) Representative hematoxylin and eosin (left panels) and Sirius red (right panels) staining. Scale bar: 100 μm. (C) Quantification of Sirius red (SR)-positive areas. Data are expressed as percentage over total field area. (D) Quantification of necroinflammatory grading using Ishak’s scoring system. (E) qPCR analysis of fibrosis and inflammation marker genes. One-way ANOVA plus Tukey’s (A and E) or Kruskal-Wallis (B and D) post-hoc test: ∗p < 0.05; ∗∗p < 0.01; ∗∗∗p < 0.005; ∗∗∗∗p < 0.0001. Data are shown as average ± SEM.

Consistent with previous reports,^10^ serum alanine and aspartate aminotransferase (ALT and AST) activities were significantly increased in *Atp7b*^−/−^ mice treated with GFP vector compared with age- and gender-matched *Atp7b*^+/−^ healthy control mice (Figure 4A). Moreover, liver histological analysis showed large areas of inflamed and fibrotic tissues with enlarged hepatocytes and nuclei (Figure 4B). In sharp contrast to control mice, int-ATP7B treatment normalized serum ALT and AST at both tested vector doses (Figure 4A) and livers of *Atp7b*^−/−^ mice injected with the higher dose of 2 × 10^13^ gc/kg of int-ATP7B showed no significant pathological changes, while reduced liver injury was observed at the lower dose of 5 × 10^12^ gc/kg (Figure 4B). Sirius red staining, grading of necroinflammatory activity, and gene expression analysis of fibrosis and inflammation marker genes confirmed the correction of liver disease by int-ATP7B (Figures 4C–4E).

**Figure 5:**
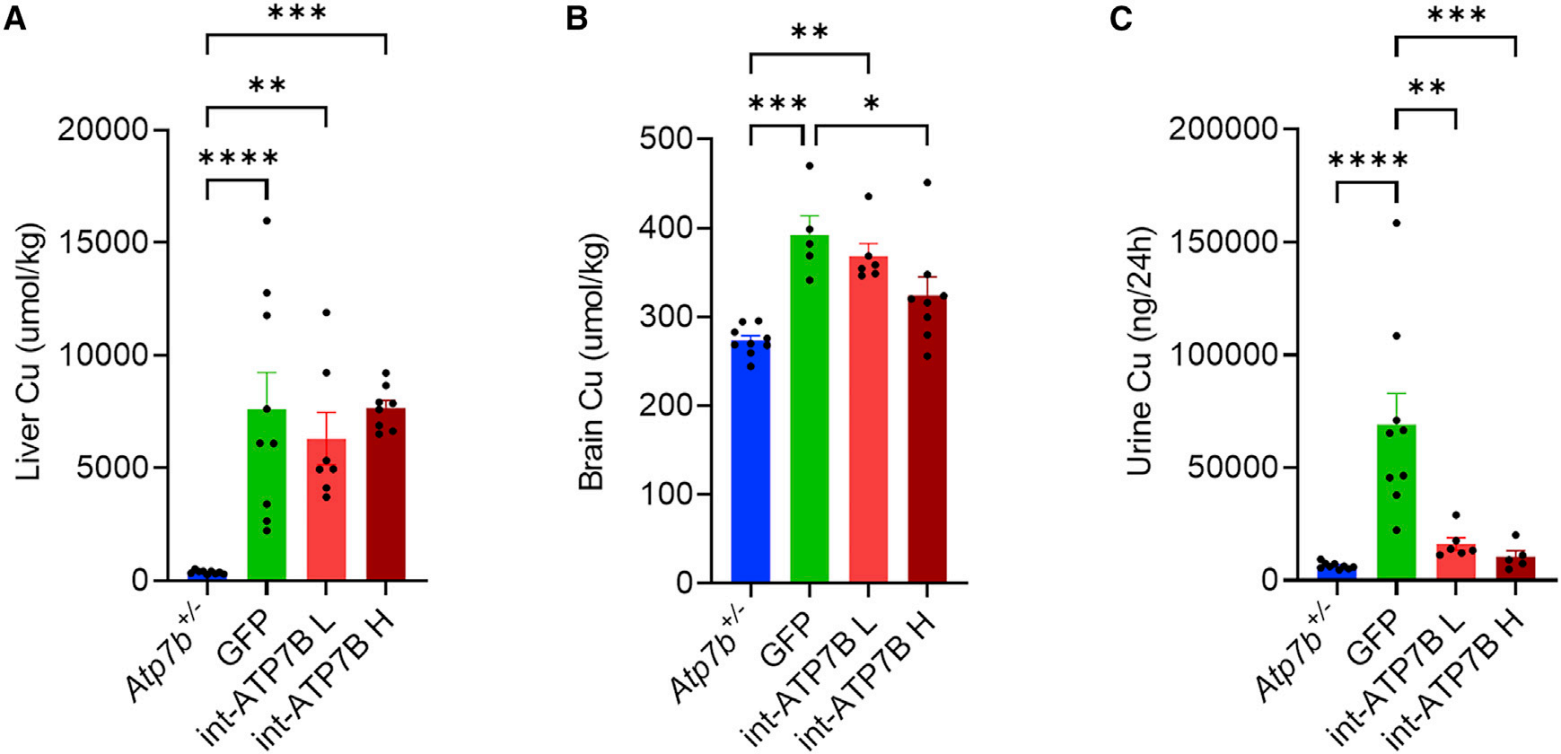
Copper content analysis Copper determination by ICP-MS in (A) liver, (B) brain, and (C) urine from *Atp7b*^+/−^ healthy control mice and *Atp7b*^−/−^ mice injected with AAV2/8-TBG-eGFP (GFP) or AAV2/8-HLP-5′ATP7B-N-intein and AAV2/8-HLP-C-intein-3′ATP7B at a dose of 5×10^12^ (int-ATP7B L) or 2×10^13^ gc/kg (int-ATP7B H). One-way ANOVA plus Tukey’s post-hoc test: ∗p < 0.05; ∗∗p < 0.01; ∗∗∗p < 0.005; ∗∗∗∗p < 0.0001. Data are shown as average ± SEM.

Although high variability was observed, hepatic copper storage was significantly increased in GFP-injected mice compared with *Atp7b*^+/−^ control mice (Figure 5A). Analysis of liver sections from mice with less severe copper storage by Timm’s sulfide silver stainingrevealed areas of regeneration characterized by strongly reduced copper storage compared with fibrotic and inflamed areas (Figure S4). Mice treated with int-ATP7B vectors showed normalization of copper levels in the brain and in the urine but not in the liver (Figures 5A–5C). Fecal copper content was similar in *Atp7b*^−/−^ and *Atp7b*^+/−^ control mice (Figure S5A), although int-ATP7B-treated *Atp7b*^−/−^ mice showed a clear increasing trend over time (Figure S5B). In summary, split intein technology effectively prevents liver disorder and reduces brain and urinary copper concentrations in WD mice.

## Discussion

Based on their efficacy and favorable safety profile, AAV vectors are currently considered the vector of choice for liver-directed gene therapy, so they have entered clinical investigation for several inherited metabolic diseases.^25^ However, limited cargo capacity represents a major hurdle to AAV-mediated delivery of genes whose coding sequence exceeds 4.0 kb. The human *ATP7B* cds spans ≈4.4 kb, and its incorporation into a standard expression cassette results in an oversized AAV genome,^10^ which is characterized by inconsistent packaging, poor production yield, and low infectivity.^26^, ^27^, ^28^ Because of these limitations, they cannot be used in clinical trials. Minimization of regulatory elements and/or mini-transgenes are strategies to circumvent this limitation. However, these strategies may affect gene expression efficiency and specificity, as well as the activity of the therapeutic protein. This approach has been applied to WD gene therapy to generate mini-ATP7Bs, which are devoid of MBD1-3 or MBD1-4, that showed efficacy *in vivo* and are currently under investigation in two independent clinical trials (NCT04537377; NCT04884815).

Dual-vector delivery of split transgene halves represents an alternative strategy to expression cassette shrinkage, and intein-mediated protein *trans*-splicing has been successfully employed to reconstitute large therapeutic proteins in the muscle,^29^ liver,^30^^,^^31^ and retina.^32^ Here, we demonstrated that split intein technology can efficiently drive reconstitution of full-length ATP7B in hepatocytes. The splitting point we selected for ATP7B fell at the N-terminus of MBD5 and did not involve essential residues for copper binding and coordination.^33^ The intein-mediated reconstitution process did not perturb ATP7B localization, trafficking, and function. Consistently, liver-directed gene transfer with int-ATP7B vectors prevented liver injury in *Atp7b*^*−/−*^ mice. Correction of liver injury did not result in reduction of hepatic copper compared to untreated animals at the tested time point. However, copper does not continuously accumulate in *Atp7b*^*−/−*^ mouse liver, but after peaking at around 2 months of age, it progressively decreases^24^ and redistributes from hepatocytes to non-parenchymal cells.^34^ This rearranging of copper deposits is thought to drive liver disease progression. Consistently, in livers from *Atp7b*^*−/−*^ mice treated with control vector, we found regenerative areas without significant pathological alteration and negligible copper storage, together with diseased liver areas accumulating copper. On this line, our findings further support the hypothesis that liver disease severity does not directly correlate with bulk copper levels in WD.^35^

While this work provided proof-of-concept for int-ATP7B efficacy in correcting WD, several issues remain toward the development of an intein-based gene therapy, that must be addressed by future studies. Int-ATP7B vectors might be tested in female and in older mice, which are more refractory to disease correction by gene therapy due to reduced hepatocyte transduction by AAV vectors. Long-term experiments are needed to investigate duration of the therapeutic effect, which may be impaired by episomal transgene dilution. Finally, a direct *in vivo* comparison of int-ATP7B and mini-ATP7B efficacy is needed to establish the most promising gene therapy candidate. Compared with mini-ATP7B delivery by a single AAV vector, the efficacy of dual int-ATP7B vectors may be limited by the need to co-transduce target cells with two AAV vectors to express the functional therapeutic product. Nevertheless, once reconstituted, full-length protein may be more active and/or stable compared with minimized protein versions.

Another drawback of dual-vector approaches may be represented by the production of truncated protein species exerting a dominant-negative effect. Although no evidence of intein-mediated toxicity has been reported so far, these concerns may be addressed by the inclusion of degradation signals (degrons) to boost proteasomal disposal of excised inteins and dominant truncated forms.^16^ Efficacy of the intein-ATP7B gene therapy approach may also be improved by the employment of smaller inteins,^36^ which would allow the incorporation of larger regulatory elements to drive stronger gene expression.

In summary, we showed that protein *trans*-splicing driven by AAV-delivered split intein machinery reconstitutes full-length human ATP7B and rescues copper-mediated liver damage in *Atp7b*^−/−^ mice, thus providing evidence of the therapeutic potential of int-ATP7B vectors as a new gene therapy approach for WD.

## Material and methods

### Plasmids

DNA constructs bearing sequences encoding codon-optimized full-length human ATP7B or ATP7B halves, split inteins from *Nostoc punctiforme* (Npu) DnaE and 3xFLAG tag were synthetized (Genewiz) and cloned into a pAAV2.1 plasmid under the control of thyroxine-binding globulin (TBG) or hybrid liver-specific (HLP) promoter for *in vitro* and *in vivo* studies, respectively. Expression cassettes also included Simian virus 40 (SV40) intron, woodchuck hepatitis virus post-transcriptional regulatory element, and bovine growth hormone polyadenylation signal (bGHpA).

### Cell studies

HepG2 ATP7B knockout cells were maintained in RPMI 1640 (Euroclone) supplemented with 10% fetal bovine serum (Euroclone) plus 1% penicillin/streptomycin solution and 1% L-glutamine (Euroclone).^23^ Cells were plated in a six-well plate and transfected using LipoD293 (SignaGen Laboratories) with a total of 2 μg of plasmid DNA per well. 72 h after transfection, cells were washed in PBS and lysed in RIPA buffer. Lysates were incubated for 30 min on ice, supplemented with protease inhibitor cocktail (Sigma), and centrifuged for 20 min. Supernatant was collected and protein content was determined by Bradford assay (Bio-Rad). Protein samples were separated by SDS-PAGE by using 4%–12% polyacrylamide gels (Bio-Rad). Primary antibody mouse anti-FLAG (Sigma-Aldrich; Cat#A8592-2MG) and mouse anti-GAPDH (Santa Cruz Biotechnology; Cat#sc-32233) were diluted in TBS-T (0.8% NaCl, 0.02% KCl, 0.3% Tris-base-0.1% Tween 20)/5% milk (Bio-Rad). Proteins of interest were detected with horseradish peroxidase (HRP)-conjugated goat anti-mouse IgG antibody (GE Healthcare). Peroxidase substrate was provided by ECL Western Blotting Substrate kit (Pierce).

To investigate Cu-dependent localization/trafficking of int-ATP7B, HepG2 ATP7B-KO cells were transfected with FLAG-tagged int-ATP7B or control wt-ATP7B cDNA constructs. The cells were then prepared for confocal microscopy directly or after exposure to either 200 μM Cu-chelating agent BCS (overnight) or 200 μM CuSO_4_ (2 h). Cells were fixed for 10 min with 4% paraformaldehyde (PFA) in 0.2 M HEPES followed by incubation with blocking/permeabilizing solution (0.5% BSA, 0.1% saponin, 50 mM NH_4_Cl in PBS) for 20–30 min. ATP7B was revealed using rabbit anti-FLAG (Sigma-Aldrich; Cat#F7425) or anti-ATP7B (Abcam; Cat#ab124973) antibodies, while Golgi or late endosomes/lysosomes were labeled with mouse anti-Golgin-97 (Thermo Fisher; Cat#A-21270) or anti-LAMP1 (Hybridoma Bank; Cat#DSHB H4A3-c) antibody, respectively. Nuclei were counterstained with Hoechst (Thermo Fisher). Samples were examined with a ZEISS LSM 700 confocal microscope equipped with a 63X 1.4 NA oil objective. Colocalization of WT- or int-ATP7B with either Golgin-97 or LAMP1 was quantified based on Pearson’s correlation coefficient using the Colocalization module of ZEISS ZEN software. ATP7B-KO cells transfected with either wt-ATP7B or int-ATP7B were treated with 0.5mM CuCl_2_ for 24 h. To analyze cell apoptosis, the cells were incubated with 6 μM CellEvent Caspase-3/7 Green Detection Reagent (Life Technologies) for 30 min, then fixed and labeled with Hoechst 33342 to counterstain nuclei. After activation of Caspase-3/7, the apoptotic cells contained nuclei of green color produced by binding of the CellEvent dye to DNA. Expression of transfected wt-ATP7B or int-ATP7B was revealed with anti-FLAG or anti-ATP7B antibodies as described above. The cells were observed using the previously mentioned confocal microscope (Zeiss LSM 700) and an appropriate filter set. The number of apoptotic nuclei positive for CellEvent reagent staining was quantified in 10 equal fields of view and expressed as a percentage of the total number of cell nuclei in ATP7B-expressing cells. Non-transfected cells in the same fields were used as a control.

### AAV vectors

Serotype 8 AAV vectors were produced by triple transfection of HEK293 cells as previously described.^37^ For Southern blot analysis, DNA was extracted from 6×10^10^ viral particles measured as genome copies (gc) for AAV2/8-HLP-5′ATP7B-N-intein, and 1.2×10^11^ gc for AAV2/8-HLP-C-intein-3′ATP7B, and for AAV2/8-HLP-full-length ATP7B. To digest unpackaged genomes, the vector solution was incubated with 30 μL of DNase I (Roche) in a total volume of 300 μL, containing 50 mM Tris (pH 7.5) and 1 mM MgCl_2_ for 2 h at 37°C. The DNase was then inactivated with 50 mM EDTA, followed by incubation at 50°C for 1 h with Proteinase K and 2.5% *N*-lauryl-sarcosyl solution to lyse the capsids. The DNA was extracted twice with phenol-chloroform and precipitated with 2 volumes of ethanol 100% and 10% sodium acetate (3 M) and 1 μL of glycogen (Roche). A total of 3×10^11^ gc for AAV2/8-HLP-full-length ATP7B and AAV2/8-HLP-C-intein-3′ATP7B, and 3×10^10^ gc for AAV2/8-HLP-5′ATP7B-N-intein were loaded on an alkaline agarose gel. A probe specific for the HLP promoter was labeled with DIG-High Prime (Roche) according to manufacturer’s protocol and used for hybridization.

### Mouse studies

Mouse procedures were carried out in accordance with the regulations and authorized by the Italian Ministry of Health. *Atp7b*^−/−^ ^38^ and C57BL/6 mice (Charles River Laboratories) were housed at TIGEM animal facility and received food and water *ad libitum*. Male mice were used for experimental procedures. AAV vectors were administered by intravenous injection in 0.9% NaCl solution to male mice at 6 weeks of age. For urine collection, mice were placed in metabolic cages for 24 h. Insoluble or suspension particles were removed by centrifugation and complete urine collection was confirmed by creatinine. At sacrifice, animals were perfused with PBS and livers were harvested for further analysis.

Western blot analysis on whole liver lysates was performed as for cell studies. For gene expression analyses, total RNA was extracted using the RNeasy mini kit (Qiagen), and 1 μg of RNA was reverse-transcribed using the High-Capacity cDNA Reverse Transcription Kit according to manufacturer’s protocol (Applied Biosystem). qPCR reactions were performed using SYBR Green Master Mix and run on a Light Cycler 480 system (Roche). Data were analyzed using LightCycler 480 software, version 1.5 (Roche Applied Science). Primer sequences are listed in Table S1. Mouse β2-microglobulin (*B2m*) was used as a housekeeping control. Serum transaminase levels were measured by scil Vitro Vet analyzer (Scil vet).

### Liver staining

Livers from PBS-perfused mice were fixed in 4% paraformaldehyde for 12 h, stored in 70% ethanol, and embedded in paraffin blocks. Hematoxylin and eosin staining was performed on 5-μm-thick paraffin sections of livers, which were rehydrated and stained in Mayer’s hematoxylin (Bio-Optica) for 4 min. After two washes in tap water for 5 min, sections were incubated in a solution of 0.1% ammonia water (1 mL ammonium hydroxide in 1 L distilled water) for 1 min, washed again in tap water for 5 min, and counterstained in eosin y-solution (Sigma) for 30 s. Sections were dehydrated, cleared in xylene, and mounted in a resinous medium. Images were captured by Axio Scan.Z1 microscope (Zeiss). Five images from each mouse were analyzed. Sections were blind evaluated by an experienced pathologist (S.C.) for necroinflammatory grading using Ishak’s scoring system.^39^

For Sirius red staining, 5-μm-thick sections were rehydrated and stained for 1 h in picrosirius red solution (0.1% Sirius red in saturated aqueous solution of picric acid). After two changes of acidified water (0.5% acetic acid in water), sections were dehydrated, cleared in xylene, and mounted in a resinous medium. Images were captured by Axio Scan.Z1 microscope (Zeiss) and analyzed using ImageJ software for quantification of Sirius red positive areas. Five images for each mouse were analyzed.

For immunohistochemistry, 5-μm-thick sections were rehydrated and permeabilized in PBS/0.2-Triton (Sigma) for 20 min. Antigen unmasking was performed in 0.01 M citrate buffer in a microwave oven. Next, sections underwent blocking of endogenous peroxidase activity in methanol/1.5% H_2_O_2_ (Sigma) for 30 min and incubated with blocking solution (3% BSA [Sigma], 5% donkey serum [Millipore], 1.5% horse serum [Vector Laboratories] 20 mM MgCl_2_, 0.3% Triton [Sigma] in PBS] for 1 h. Sections were incubated with primary antibody (rabbit anti-ATP7B, Thermo Fisher Scientific, Cat#PA-102826, dilution: 1/100) overnight at 4°C and then with universal biotinylated horse anti-mouse/rabbit IgG secondary antibody (Vector Laboratories) for 1 h. Biotin/avidin-HRP signal amplification was achieved using the ABC Elite Kit (Vector Laboratories) according to manufacturer’s instructions. 3,3′-diaminobenzidine (Vector Laboratories) was used as the peroxidase substrate. Mayer’s hematoxylin (Bio-Optica) was used for counter-staining. Sections were de-hydrated and mounted in Vectashield (Vector Laboratories). Image capture was performed using Axio Scan.Z1 microscope (Zeiss).

For Timm’s staining, 5-μm-thick sections were rehydrated and incubated sequentially in 0.5% ammonium sulfide (5 min), deionized water (1 min rinse), 0.1 N HCl (2–3 min), deionized water (2–3 min rinse), and developer (10 min). The developer was made of 1 part 5% silver nitrate and 5 parts of a solution consisting of 2% w/v hydroquinone and 5% w/v citric acid. Sections were finally washed in deionized water for 1 min, counterstained with Harris hematoxylin solution (Bio-Optica), dehydrated, and mounted in resinous medium. Images were captured by Axio Scan.Z1 microscope (Zeiss).

### Copper measurement

For determination of copper content, liver, brain, and feces samples were dried to constant weight in a Savant Speedvac centrifugal concentrator (Thermo Fisher). Samples were digested using an “open vessel” method. All sample containing tubes and two blank tubes were punctured on the lid to avoid pressure build-up during the procedure. To each tube, 0.2 mL of Trace Metal Grade concentrated nitric acid (A509 Trace Metal Grade; Fisher) containing (5% v/v) Agilent Internal Standard mixture (5183-4681; Agilent Technologies) was added. Tubes were then inserted into a “Dri-block” heater at room temperature, set to 60°C for the first 30 min and 100°C for a further 210 min. After digestion, the tubes were allowed to cool to room temperature, and 100-μL aliquots were taken from each digestion solution and added to 15-mL Falcon tubes containing 5 mL LC-MS grade water, to produce solutions for analysis at a final nitric acid concentration of 2% (v/v). Urine samples were not digested. Samples were diluted 200-fold in 2% nitric acid containing internal standards.

Copper concentrations were measured using an Agilent 7700x ICP-MS spectrometer equipped with a MicroMist nebulizer (Glass Expansion) and a Scott double-pass spray chamber. Nickel sample and skimmer cones were used. Sample introduction was performed using an Agilent Integrated autosampler (I-AS). A multi-element method including all elements present in the calibration solution, as previously reported,^40^ was applied. Calibration solutions were produced by appropriate dilutions of Environmental Calibration Standard (Agilent 5183-4688). Helium was used as collision gas; copper levels were analyzed in helium (He) mode (5.0 mL min-1 helium) with an integration time of 0.3 s. For each analytical batch, multi-element calibration was performed using serial dilutions of the calibration standard. An intermediate concentration from this calibration series was used as a periodic quality control sample throughout each analytical batch. Instrument and digestion blanks were also interspersed through each set of randomized samples.

### Statistical analyses

Statistical analyses were performed using GraphPad Prism software. One-way ANOVA plus Tukey’s or Kruskal-Wallis post-hoc test was used as statistical test for mean comparison. Experimental group sizes are reported in the figures. Data are shown as average ± SEM.

## Supplemental information

Document S1. Figures S1–S5 and Table S1

Document S2. Article plus supplemental information
