# Supplementary material for: Full-length ATP7B reconstituted through protein trans-splicing corrects Wilson disease in mice
Source: Mol Ther Methods Clin Dev. 2022 Aug 13;26:495–504. doi: 10.1016/j.omtm.2022.08.004 (PMC9436707; doi:10.1016/j.omtm.2022.08.004)
Supplement: Document S1. Figures S1–S5 and Table S1 [file mmc1.pdf]

OMTM, Volume *26*

Supplemental information

Full-length ATP7B reconstituted through protein *trans*-splicing corrects Wilson disease in mice

Agnese Padula, Raffaella Petruzzelli, Sasha A. Philbert, Stephanie J. Church, Federica Esposito, Severo Campione, Marcello Monti, Filomena Capolongo, Claudia Perna, Edoardo Nusco, Hartmut H. Schmidt, Alberto Auricchio, Garth J.S. Cooper, Roman Polishchuk, and Pasquale Piccolo

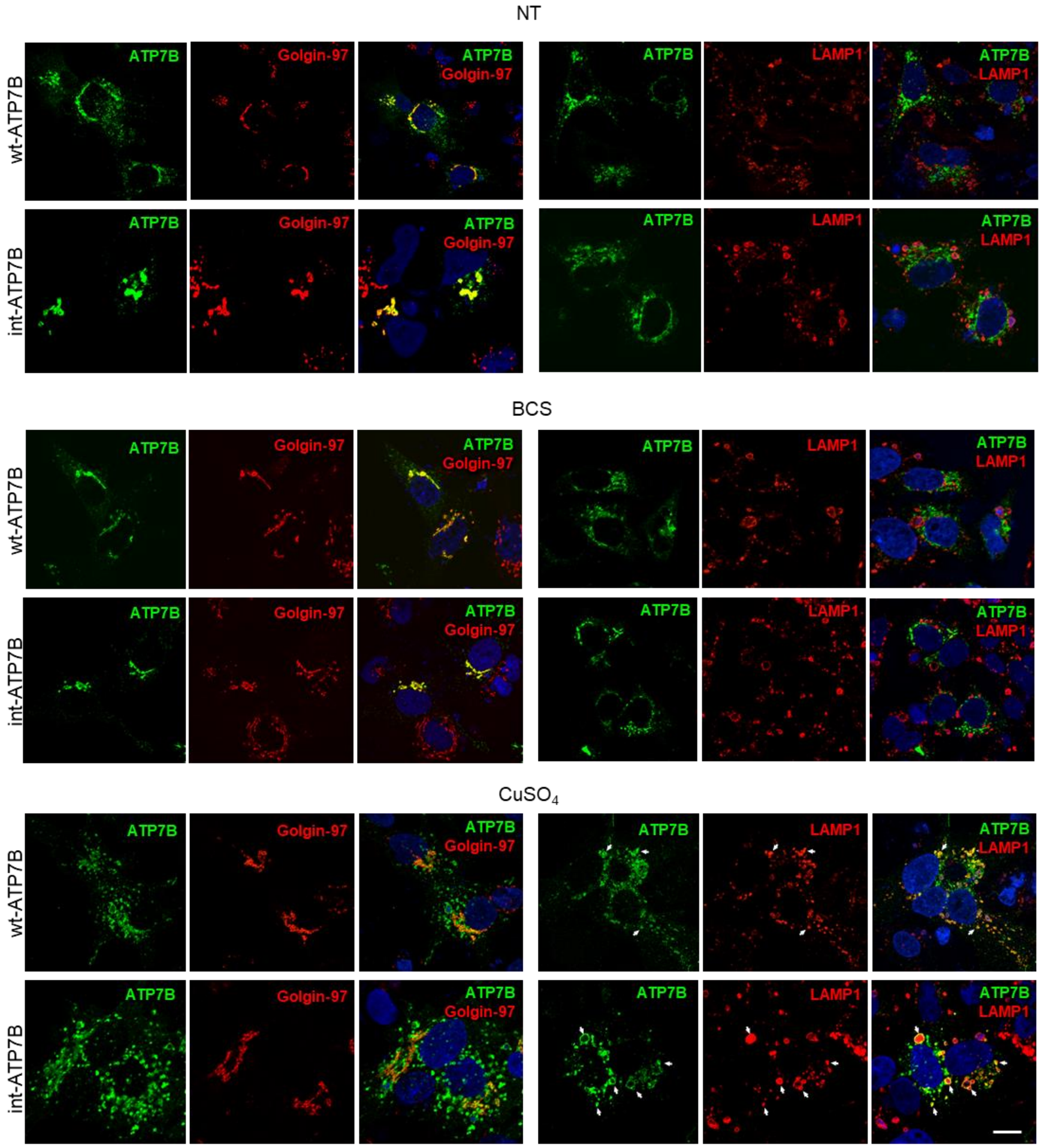

Figure S1. Representative images from immunofluorescent staining of ATP7B-KO HepG2 cells transfected with wt-ATP7B or int-ATP7B constructs and left untreated (*higher panels*), or after treatment with copper chelator bathocuproine disulphonate (BCS, *center panels*) or with CuSO4 (*lower panels*). Cells were labeled with antibodies against Golgin-97 and LAMP1 (red), and anti-ATP7B or anti-FLAG antibody to detect ATP7B (green). Scale bar: 10µm

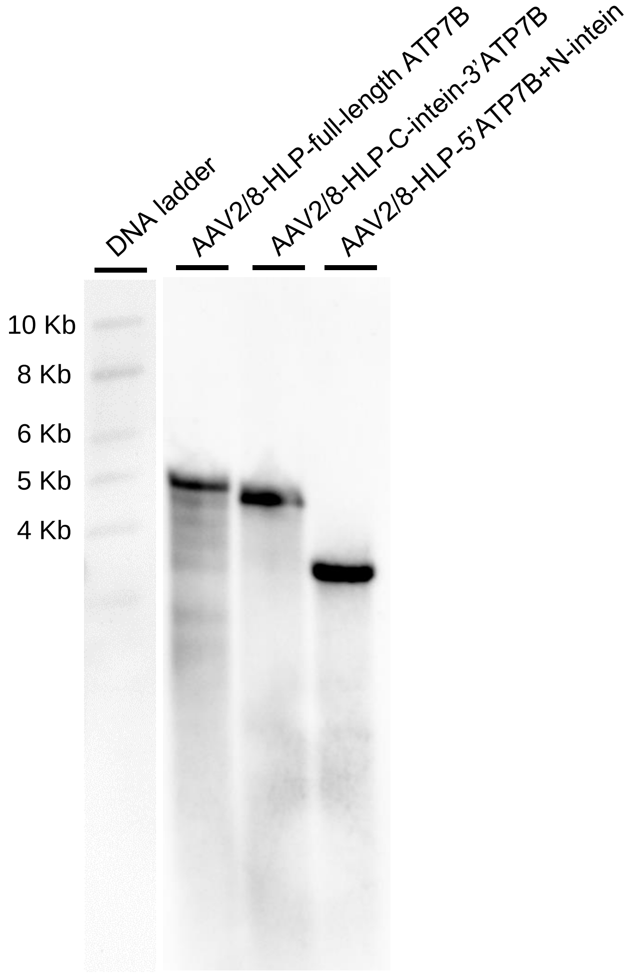

Figure S2. Alkaline gel Southern blot analysis of genomic DNA from AAV preparations. AAV DNA was hybridized to a probe specific for the HLP promoter. Expected genome sizes: AAV2/8-HLP-full-length ATP7B, 5.0kb; AAV2/8-HLP-C-intein-3'ATP7B, 4.7kb; AAV2/8-HLP-5'ATP7B+N-intein, 3.5kb.

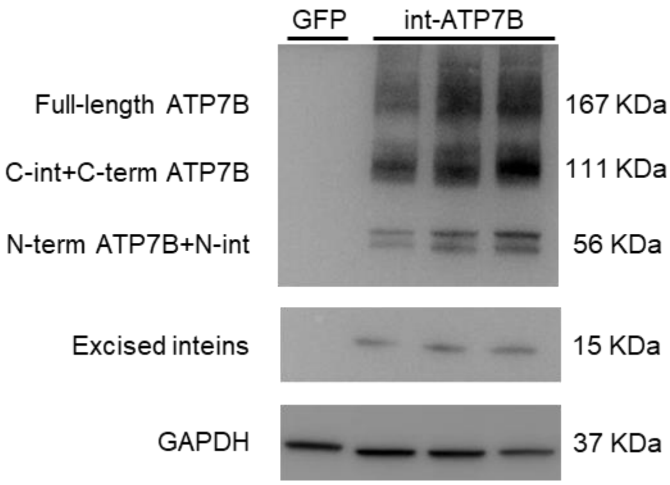

Figure S3. Western blot analysis using anti-FLAG antibody of whole liver lysate from wild-type mice injected with AAV2/8-TBG-eGFP (GFP) at a dose of $2x10^{13}$gc/Kg or co-injected with AAV2/8-HLP-5'ATP7B+N-intein and AAV2/8-HLP-C-intein-3'ATP7B (int-ATP7B), at a total dose of $2x10^{13}$gc/Kg. Expected molecular weights are: 167 kDa for full-length ATP7B-3XFLAG, 111kDa for C-intein-c-term ATP7B half-3XFLAG, 56 kDa for N-term ATP7B half-N-intein-3XFLAG, and 15kDa for excised inteins. GAPDH was used as loading control.

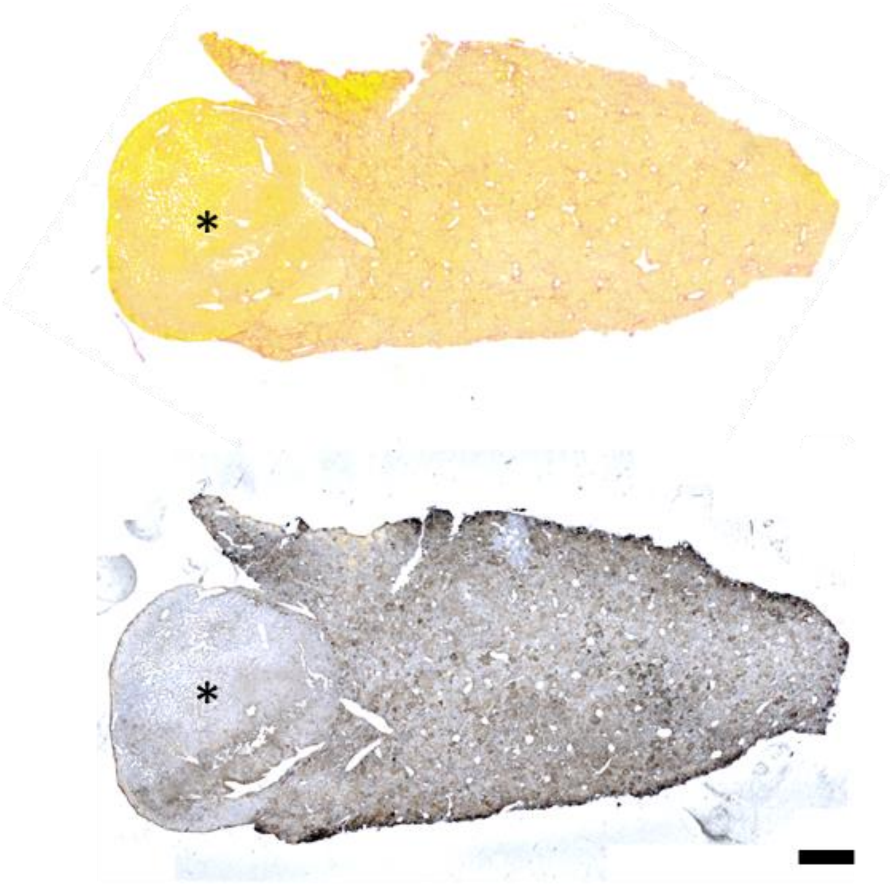

Figure S4. Representative images from Sirius Red (*upper panel)* and Timm's silver *(lower panel)* staining on serial liver sections from *Atp7b*$^{-/-}$ mouse injected with AAV2/8-TBG-GFP showing low hepatic copper accumulation (see **Fig. 5A**) Regenerative area is indicated by asterisks. Scale bar: 500µm.

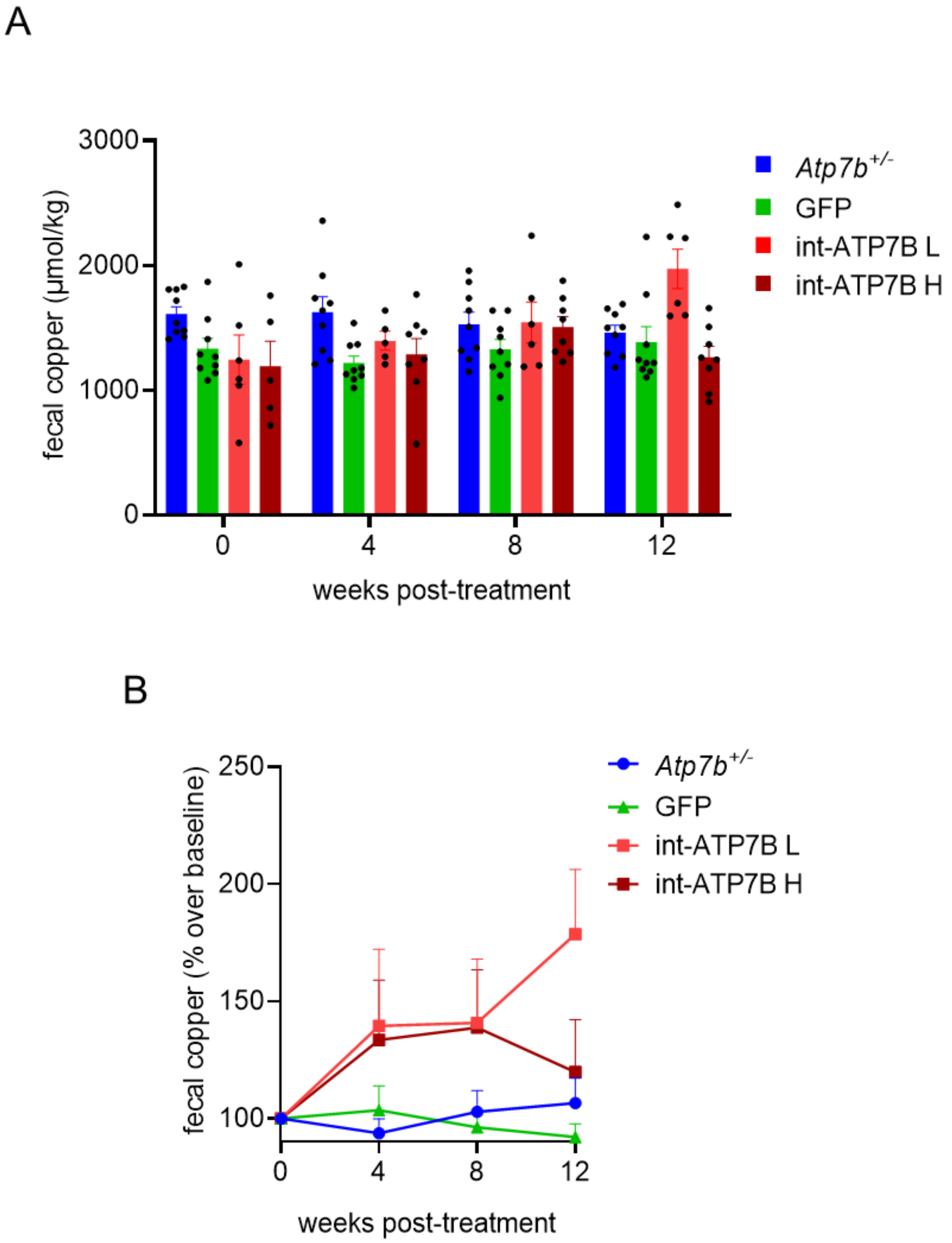

Figure S5. **A)** Copper determination by ICP-MS in feces from *Atp7b*$^{+/-}$ healthy control mice and *Atp7b*$^{-/-}$ mice injected with AAV8-TBG-eGFP (GFP) or AAV8-HLP-5’ATP7B-N-intein and AAV8-HLP-C-intein-3’ATP7B at a dose of $5x10^{12}$ (int-ATP7B L) or $2x10^{13}$ gc/kg (int-ATP7B H). **B)** Fecal copper content expressed as percentage over baseline

Table S1. Primers used for qPCR analysis

Gene	Forward (5'-3')	Reverse (5'-3')
Acta2	GACGTACAACTGGTATTGTGC	CTCGGCAGTAGTCACGAAGG
B2m	TGGTGCTTGTCTCACTGACC	GTATGTTCGGCTTCCCATTC
Ccl2	GCTCAGCCAGATGCAGTTAA	TCTTGAGCTTGGTGACAAAAACT
Col1a1	GCCAAGAAGACATCCCTGAA	GCCATTGTGGCAGATACAGA
Timp1	CTCATCACGGGCCGCCTAAG	CACTGTGCACACCCCACAGC
